# Temporal Roles of Platelet and Coagulation Pathways in Collagen- and Tissue Factor-Induced Thrombus Formation

**DOI:** 10.3390/ijms23010358

**Published:** 2021-12-29

**Authors:** Stefano Navarro, David Stegner, Bernhard Nieswandt, Johan W. M. Heemskerk, Marijke J. E. Kuijpers

**Affiliations:** 1 Institute of Experimental Biomedicine I, University Hospital Würzburg, Würzburg Josef-Schneider-Straße 2, 97080 Wurzburg, Germany; Navarro_S@ukw.de (S.N.); stegner@virchow.uni-wuerzburg.de (D.S.); bernhard.nieswandt@virchow.uni-wuerzburg.de (B.N.); 2 Rudolf Virchow Center for Integrative and Translational Bioimaging, University of Würzburg, 97080 Wurzburg, Germany; 3 Department of Biochemistry, Cardiovascular Research Institute Maastricht (CARIM), Maastricht University, 6229 ER Maastricht, The Netherlands; 4 Synapse Research Institute, Kon. Emmaplein 7, 6214 KD Maastricht, The Netherlands; 5 Thrombosis Expertise Center, Heart and Vascular Center, Maastricht University Medical Center+, Maastricht, Professor Debyelaan 25, 6229 HX Maastricht, The Netherlands

**Keywords:** coagulation, fibrin, glycoprotein VI, platelet receptors, spatiotemporal thrombus, thrombin

## Abstract

In hemostasis and thrombosis, the complex process of thrombus formation involves different molecular pathways of platelet and coagulation activation. These pathways are considered as operating together at the same time, but this has not been investigated. The objective of our study was to elucidate the time-dependency of key pathways of thrombus and clot formation, initiated by collagen and tissue factor surfaces, where coagulation is triggered via the extrinsic route. Therefore, we adapted a microfluidics whole-blood assay with the Maastricht flow chamber to acutely block molecular pathways by pharmacological intervention at desired time points. Application of the technique revealed crucial roles of glycoprotein VI (GPVI)-induced platelet signaling via Syk kinase as well as factor VIIa-induced thrombin generation, which were confined to the first minutes of thrombus buildup. A novel anti-GPVI Fab EMF-1 was used for this purpose. In addition, platelet activation with the protease-activating receptors 1/4 (PAR1/4) and integrin αIIbβ3 appeared to be prolongedly active and extended to later stages of thrombus and clot formation. This work thereby revealed a more persistent contribution of thrombin receptor-induced platelet activation than of collagen receptor-induced platelet activation to the thrombotic process.

## 1. Introduction

In hemostasis, thrombosis, and thrombo-inflammation, multiple platelet and coagulation activation processes interact to establish the formation of a thrombus composed of aggregated and contracted platelets connected by a fibrin network or clot [1,2,3]. Consolidation of the thrombus is considered to be regulated by the release of paracrine platelet agonists and by local thrombin activity [4,5]. In the past years, several key molecular actors of the thrombus-forming process have been identified. The first interaction of platelets with exposed vascular collagen occurs via glycoprotein Ib-V-IX (GPIb)-dependent platelet adhesion to collagen-bound von Willebrand factor (VWF). The initial adhesion facilitates the interaction of glycoprotein VI (GPVI) with collagen. Mouse thrombosis models have confirmed the key role of platelet GPVI as a signaling collagen receptor, acting via protein tyrosine kinases such as Syk and culminating in the activation of phospholipase Cγ2 and phosphoinositide 3-kinases [6,7,8,9]. Recently, the key role of GPVI in thrombus formation was confirmed by studies with blood from patients with a congenital deficiency in the gene *GP6* [10]. Both in mouse and human, GPVI-induced signaling leads to platelet granule release, activation of integrin αIIbβ3, platelet shape change, and surface exposure of the procoagulant phospholipid phosphatidylserine [11,12]. For a stable platelet adhesion to collagen, in addition, binding via the integrins α2β1 and αIIbβ3 is required [6]. In thrombosis and hemostasis, the binding of fibrinogen to activated integrin αIIbβ3 on adjacent platelets generates a scaffold for the formation of platelet aggregates and, in flowing blood, for the buildup of a thrombus [2,11,13].

Regarding the initiation of coagulation, vascular exposed tissue factor (TF) acts as an initial trigger of the extrinsic pathway and stimulates the proteolytic coagulation cascade of factor VIIa, factor Xa and thrombin [14,15,16,17]. The exposed phosphatidylserine on highly activated platelets enhances the generation thrombin to produce sufficient amounts for the formation of a fibrin clot [12,18,19,20]. It is stipulated [21] but incompletely studied that the generated thrombin stimulates human platelets by targeting the PAR1 and PAR4 receptors. Additional platelet activation is achieved by platelet-derived autocoids, which enhance the activation of αIIbβ3 and thus mediate further thrombus growth [22,23]. The thrombin-dependent formation of fibrin fibers consolidates the thrombus, converting it into a vessel-occlusive clot.

Using microfluidic approaches, we and others have shown that immobilized TF in a dose-dependent way enhances the platelet activation in thrombus formation and triggers the formation of a fibrin clot [24,25,26]. To investigate the molecular pathway in that process, we developed and validated a microfluidics test, operating in combination with multicolor microscopy, which provided multiparameter information on the formation of platelet-fibrin thrombi during whole-blood perfusion [26]. These microfluidic studies showed that collagen-induced GPVI signaling led to thrombi with patches of phosphatidylserine-exposing platelets, which acted as a driving force for ensuing thrombin and fibrin generation [27,28]. Recent studies also revealed an intricate molecular synergy between the actions of immobilized collagen and TF, in that TF alone was unable to support platelet adhesion, while collagen alone was a poor supporter of the clot-forming process [26]. However, the precise time frame by which TF and collagen, and by consequence the molecular actions of PARs and GPVI operate, has remained unclear. Knowledge of this is becoming important with the new finding that GPVI also mediates platelet adhesion and activation to fibrin [29,30,31].

In recent years, many studies have sought to unravel the molecular and biochemical mechanisms leading to an optimal hemostatic response after vascular damage and to provide insights into the spatiotemporal regulation of platelet activation, aggregation, coagulation, and thrombus formation using, for instance, in vivo imaging [32] and mathematical modeling [33]. It is then silently assumed that the various platelet receptors and their downstream signaling pathways are in continuous operation during the whole period of thrombus buildup and fibrin clotting. However, if this is really the case is yet to be elucidated.

For the present paper, we hypothesized that the molecular pathways of flow-dependent platelet activation during the formation of a thrombus and clot are time-restricted. On the basis of prior experiments, we considered that the first two min of flow are critical for an initial stage of thrombus formation [26]. To investigate the time-dependent role of these pathways in more detail, we developed a procedure using the Maastricht flow chamber, in which during continuous blood flow specific inhibitors could be introduced acutely at a requested time point, typically after 2 min of start. To do so, we modified a previously standardized microfluidic device containing collagen and collagen/TF microspots [34]. By real-time microscopic examination of platelet adhesion, platelet aggregation, and fibrin formation under high-shear flow, we then sought to distinguish between early (<2 min) and later (2–10 min) contributions of the examined pathways relevant for thrombus formation at arterial flow conditions.

## 2. Results

### 2.1. Time-Restricted Roles of Tissue Factor and Factor VIIa in Collagen-Dependent Formation of Platelet-Fibrin Thrombi

In order to distinguish between early and late contributions of the thrombus pathways of interest, we needed to adapt the earlier used microfluidic system. Changes entailed the insertion of a triple inlet tubing system, where each of the three inlet tubes was connected to a 1 mL plastic syringe, the first of which contained recalcification medium, the second untreated blood, and the third inhibitor-treated blood (see Section 5). The flow perfusion rate with each syringe was controlled with a pulse-free nanopump. The tubing system and the precise flow chamber inlet dimensions allowed for an instant and complete mixing of (control or inhibited) blood with the recalcification medium. By using this three-way inlet system, we thus could keep a continuous blood flow during the change from control to inhibited blood samples, for which change was by default achieved by switching between pumps 2 and 3 after 2 min of initial blood flow.

After checking of proper functioning of the triple inlet system, i.e., the complete blood mixing with recalcification medium (data not shown), we performed a series of experiments where blood samples were flowed over microspot pairs of collagen and collagen/TF at a final shear rate of 1000 s^−1^. The two blood samples with or without inhibitor were equally pre-labeled with DiOC_6_ to detect adhered platelets, with Alexa Fluor (AF)568 annexin A5 to identify phosphatidylserine-exposing platelets, and with AF647-fibrinogen to detect fibrin formation, as detailed previously [26]. Following this process, at 2 min intervals, brightfield and multicolor fluorescence microscopic images were taken from each microspot. The images were analyzed in a standard way to produce a total of seven platelet, thrombus, and coagulation parameters (P1-7) per time point and per surface type (Table 1).

**Figure 1:**
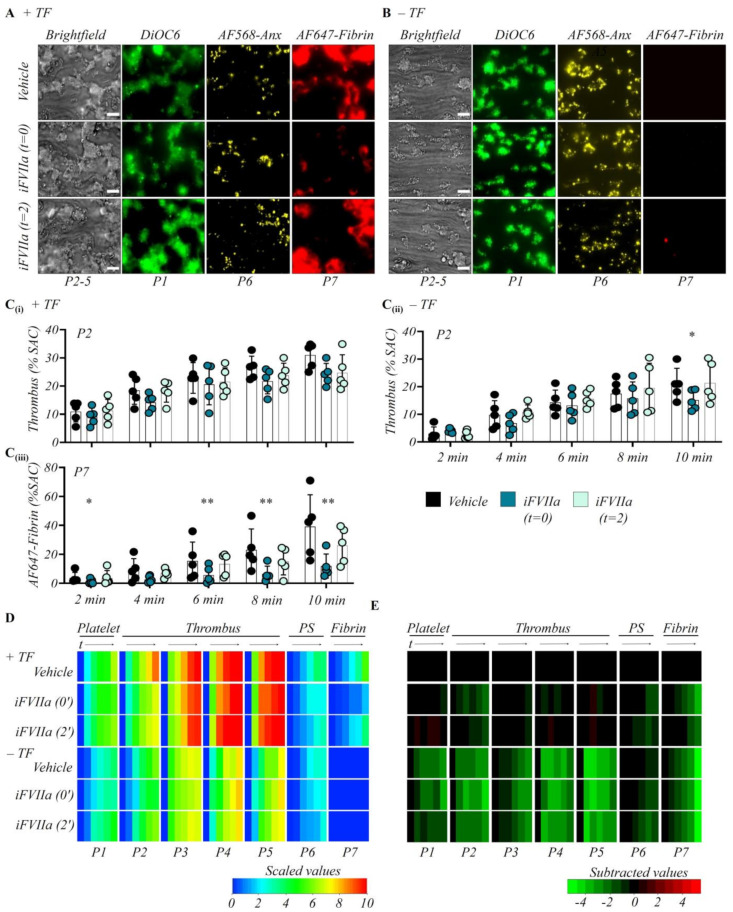
Early contributions of TF and factor VIIa in whole-blood thrombus formation. Citrated whole blood samples from healthy subjects (*n* = 5) were supplemented with fluorescent labels to simultaneously detect platelet adhesion (P1, DiOC_6_), thrombus and platelet multilayer characteristics (P2–5, brightfield), phosphatidylserine exposure (P6, AF568-annexin A5), and fibrin deposition (P7, AF647-fibrin). Using a three-way tubing inlet system, allowing complete fluid mixing, we co-infused blood samples with recalcification medium and perfused them through a parallel-plate flow chamber containing microspots of collagen (upstream) and collagen/TF (downstream) at a wall-shear rate of 1000 s^−1^. During blood flow, monochromatic images in 4 colors were captured by brightfield and fluorescence microscopy at 2, 4, 6, 8 and 10 min. Where indicated, the perfusion was using iFVIIa-treated blood (1 µM, f.c.) from the start (t = 0 min), or the iFVIIa-treated blood was introduced after 2 min. Control runs were carried out with blood samples containing vehicle solution. (**A**,**B**) Representative 10-min end-stage images of vehicle control, early extrinsic pathway inhibition (iFVIIa from start), and later extrinsic pathway inhibition (iFVIIa from 2 min). Images were from collagen microspots in the presence (**A**) or absence (**B**) of TF. Bars = 20 μm. Quantitative analysis from collagen ± TF surfaces of parameter P2: thrombus coverage (**Ci**,**Cii**) and P7: fibrin deposition (**Ciii**). Fibrin staining was essentially absent on collagen-only microspots. Means ± SD, * *p* < 0.05, ** *p* < 0.01 vs. indicated group (*t*-test). (**D**) Heatmap of univariate scaled values per parameter P1–7 for indicated surfaces and conditions. (**E**) Subtraction heatmap of scaled parameters versus vehicle control (collagen/TF), filtered for relevant changes with *p* < 0.05; color codes as indicated in color bars. For additional dataset, see Figure S1.

As usual, the perfusion of control (vehicle) blood over collagen/TF microspots resulted in fast platelet adhesion, followed by platelet aggregation and formation of contracting thrombi. The thrombi contained phosphatidylserine-exposing platelets, which at later time points were surrounded by fibrin fibers (Figure 1A). In the absence of TF, smaller and less contracted platelet aggregates were formed, while essentially no fibrin was seen during the first 10 min (Figure 1B). These observations were in agreement with previous findings [26]. Quantification of the images from the collagen/TF surface, taken over time, showed a consistent and gradual increase in thrombus coverage (P2) and in fibrin deposition (P7), starting at around 4 min (Figure 1C,D). In the absence of TF, the process of thrombus formation was slower in onset. Heatmap representation of the univariate scaled parameters P1-7 up to 10 min illustrated a decelerating effect on collagen-only microspots for essentially all parameters, except for phosphatidylserine exposure (Figure 1D). This was also concluded from a subtraction heatmap of the scaled parameters vs. those of collagen/TF microspots (vehicle condition) (Figure 1E). Together, this analysis pointed to an overall stimulating role of TF on platelet deposition, activation, and aggregation, as well as on thrombus consolidation and fibrin clotting.

It is well known that TF triggers the clotting by forming a complex with factor VII(a) and factor Xa, which mediates the generation of more factor Xa and thrombin [11]. For the microspots of collagen/TF, we investigated the time-dependency of this role of TF by treating the blood with inhibited factor VIIa (iFVIIa). In the experiments, iFVIIa was either present from the start, or was introduced after 2 min by pump switching. The chosen concentration of 1 µM iFVIIa has been shown to block all clotting-related TF activity under high-shear flow conditions [26].

For collagen/TF microspots, the initial presence of iFVIIa insignificantly reduced thrombus coverage (P2) and other thrombus parameters, while it completely and significantly suppressed fibrin formation to a level resembling that of collagen-only surfaces (Figure 1A–D). The iFVIIa effects were clearly apparent from a subtraction heatmap presentation of all parameters P1-7 over time (Figure 1E). Markedly, the inclusion of iFVIIa after 2 min did no longer influence any of the thrombus and fibrin parameters (Figure 1D,E). In comparison, for the collagen-only surfaces, the early presence of iFVIIa (0 min) only had a significant reducing effect on thrombus coverage at the latest time point, while the iFVIIa did not affect platelet adhesion and phosphatidylserine exposure (Figure S1). Moreover, the late presence of iFVIIa was without effect here. Together, these collagen/TF data pointed to a time-restricted role of the TF-factor VIIa-driven coagulation pathway in thrombus consolidation and fibrin deposition. A residual effect of iFVIIa in the absence of coated TF has been noticed before and is explained by redundancy between the intrinsic and extrinsic pathways of thrombin generation [26].

### 2.2. Longer-Term Roles of Receptors PAR1 and PAR4 in Formation of Platelet-Fibrin Thrombi

Earlier data with collagen-only microspots pointed to a slow onset of thrombin generation via the intrinsic pathway [26]. Given the high responsiveness of platelets to thrombin acting via Gqα-protein-coupled receptors PAR1 and PAR4 [35], we then unraveled the time-dependency of this thrombin receptor pathway. For this purpose, we blocked both PAR1 and PAR4 using 2 µM atopaxar (f.c.) and 1 µM BMS-986120 (f.c.), respectively. Using whole-blood flow cytometry, we checked that these doses were optimal for suppressing thrombin-induced platelet activation (J. Zou, unpublished data, 2021).

**Figure 2:**
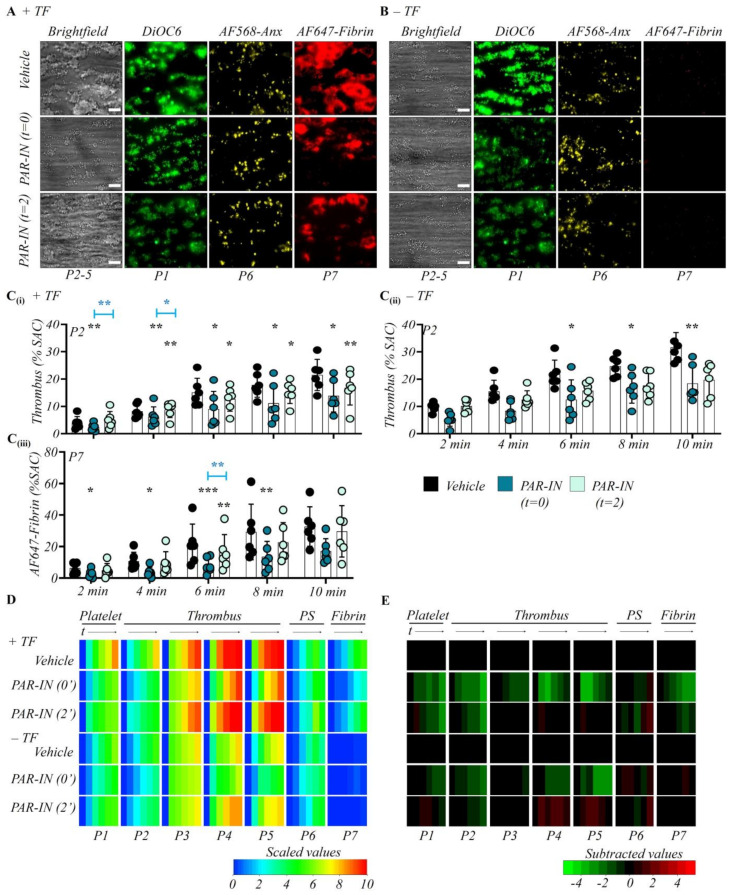
Involvement of platelet thrombin receptors PAR1 and PAR4 in thrombus formation. Citrated whole-blood samples from healthy subjects (*n* = 6) were supplemented with fluorescent labels and perfused over microspots of collagen and collagen/TF, as shown in Figure 1. Where indicated (PAR-IN), perfusion was switched from control blood to blood preincubated with vehicle or a mix of atopaxar (PAR1 inhibitor, 2 µM, f.c.) and BMS-986120 (PAR4 inhibitor, 1 µM, f.c.). Control blood runs were carried out with vehicle solution. Thrombus formation on both microspots was analyzed from captured images for parameters P1-7, as in Figure 1. (**A**,**B**) Representative 10 min end stage images of vehicle control condition; early PAR-IN (mix from start); later PAR-IN (mix from 2 min). Images were taken from microspots in the absence (**A**) or presence (**B**) of TF. Quantitative analysis from ± TF surfaces of parameter P2: thrombus coverage (**Ci**,**Cii**), and P7: fibrin deposition (**Ciii**). Means ± SD, * *p* < 0.05, ** *p* < 0.01, *** *p* < 0.001 vs. indicated group (*t*-test). (**D**) Heatmap of univariate scaled time-dependent values of P1–7 for indicated surfaces and conditions. (**E**) Subtracted heatmap of scaled parameters versus collagen and collagen/TF control runs, further as for Figure 1. For additional data, see Figure S2.

For the collagen/TF surfaces, initial blockage of PAR1+4 resulted in substantial and significant decreases in platelet adhesion (P1), thrombus growth (P2), and fibrin formation (P7) (Figure 2A–E). In contrast, phosphatidylserine exposure (P6) was unaltered (Figure S3A,C). Regarding thrombus contraction (P4) and multilayering (P5), the early PAR1+4 inhibition caused marked and significant reductions over time, approaching the thrombus parameters in the absence of TF (Figure S3E,G).

A different pattern of changes was obtained when PAR1+4 were inhibited after 2 min. On collagen/FT, the later intervention continued to reduce the thrombus growth (Figure 2A,C), but the thrombus characteristics (P3–5) remained unaltered. Markedly, fibrin formation (P7) was still moderately reduced at 6–8 min, which was linked to the reduction in thrombus size (Figure 2C).

Regarding collagen-only microspots, initial inhibition of the PAR1+4 caused a reduction of platelet adhesion (P1), thrombus coverage (P2), and other thrombus characteristics (P4–5), which was seen at all time points but was not always significant (Figure 2B–E). No such effects were seen upon late PAR1+4 inhibition (Figure 2E). 

Similarly, no effect was detected on platelet phosphatidylserine exposure (Figure S2). Interestingly, these effects of PAR1+4 inhibition mostly phenocopied the effects of absence of TF. Jointly, the results indicated a role of PAR1+4 on initial thrombus progression and contraction, especially in the presence of TF, and hence pointed to a most pivotal role of thrombin-induced signaling in the early phase of thrombus formation, but with a residual effect at later stages of the process. This implies a continued co-operation between coagulation pathways and platelet activation processes during thrombus growth.

### 2.3. Initial Contribution of GPVI and Downstream Tyrosine Kinase Signaling in Formation of Platelet-Fibrin Thrombi

**Figure 3:**
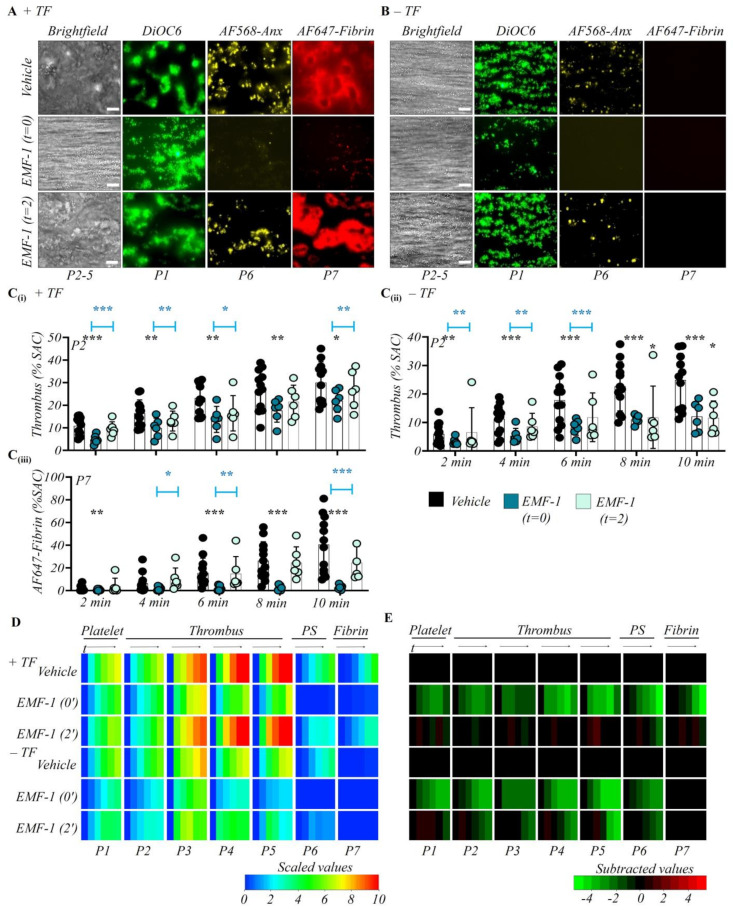
Time-dependent contribution GPVI in thrombus formation. Citrated whole blood was labeled and co-perfused with recalcification medium over collagen and collagen/TF microspots (*n* = 6). Where indicated, perfusion was switched from control blood to blood preincubated with vehicle or anti-GPVI Fab EMF-1 (10 µg/mL, f.c.). Thrombus parameter analysis and heatmap presentation were as for Figure 1. (**A**,**B**) Representative end stage images of vehicle control condition; early GPVI inhibition (EMF-1 Fab from start); later GPVI inhibition (EMF-1 Fab from 2 min). Images from microspots without (**A**) or with (**B**) TF; bars = 20 μm. Quantitative analysis from ± TF surfaces of parameter P2: thrombus coverage (**Ci**,**Cii**), and P7: fibrin deposition (**Ciii**). Means ± SD, * *p* < 0.05, ** *p* < 0.01, *** *p* < 0.001 vs. indicated group (*t*-test). (**D**) Heatmap of univariate scaled time-dependent values, and (**E**) subtracted heatmap of scaled parameters versus control runs, as for Figure 1. For additional data, see Figure S3.

We subsequently investigated the early and later roles of collagen receptor GPVI in thrombus formation using the same intervention setup. To functionally block GPVI, we used a novel, recently characterized anti-human GPVI Fab fragment EMF-1 at optimal concentration of 10 µg/mL (Emfret Analytics Ltd., Würzburg, Germany, unpublished data, B. Nieswandt, June 2021). For collagen/TF surfaces, the initial GPVI blockage led to a formation of smaller and unstable thrombi when compared to the vehicle control condition (Figure 3A). A similar change was observed for collagen-only surfaces (Figure 3B). On either surface type, platelet adhesion (P1) was partly reduced, while phosphatidylserine exposure (P6) was almost completely annulled (Figure S3A–D). These findings agreed with the understanding that exposure of phosphatidylserine is a marker of collagen-induced GPVI signaling [36]. Quantitative image analysis revealed that the blockage of GPVI with EMF-1 Fab from start led to a robust reduction of the majority of platelet activation and thrombus parameters (P2-6) on collagen ± TF surfaces (Figure 3C–E). In the presence of TF, fibrin deposition (P7) was abolished as well (Figure 3), which agreed with the procoagulant effect of phosphatidylserine exposure in the flow assay [26].

We then determined how GPVI blockage with EMF-1 Fab influenced the process, when introduced after 2 min of perfusion. In the absence of TF, this late intervention led to a moderate reduction in ensued thrombus formation. With TF present, fibrin clotting was unaffected (Figure 3C). Heatmap-based analysis showed that the thrombus parameters (P2–5) were moderately reduced in the absence of TF (Figure 3D,E). To a certain extent, the reduction in phosphatidylserine exposure also persisted (Figure S3C,D). Together, this pointed to a mostly early pivotal role of GPVI-induced platelet activation in thrombus formation and clotting. At later phase, residual GPVI-mediated effects could apparently be taken over by TF, and hence thrombin generation.

**Figure 4:**
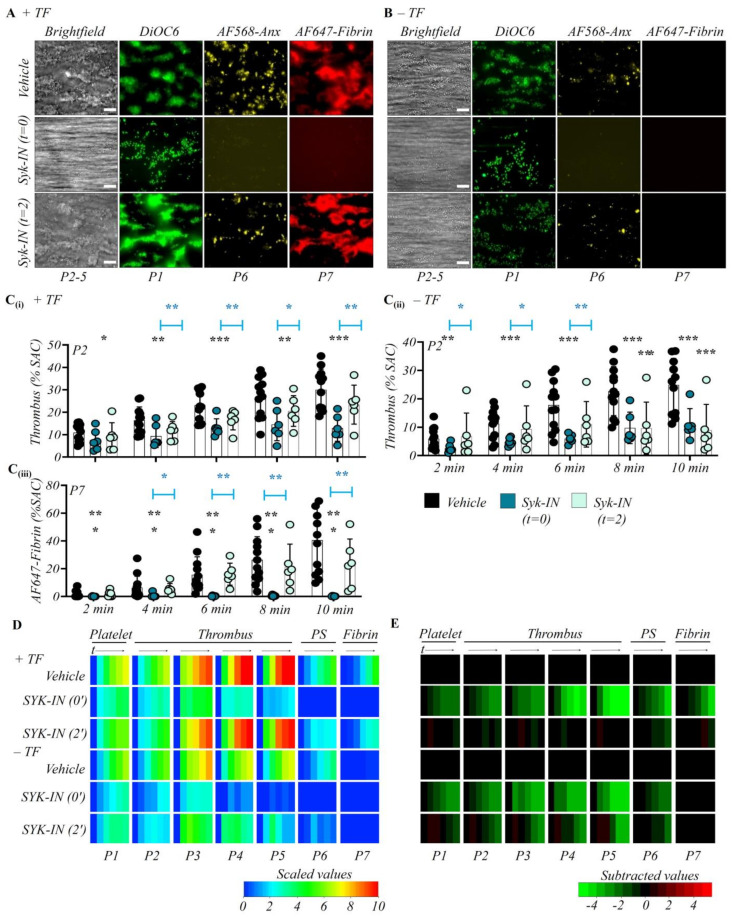
Time-confined role of Syk tyrosine kinase in thrombus formation. Citrated whole blood was labeled and co-perfused with recalcification medium over collagen and collagen/TF microspots (*n* = 6), as for Figure 1. Where indicated, perfusion was switched from control blood to blood preincubated with vehicle or Syk-IN (PRT-060318, 20 μM). Thrombus parameter analysis and heatmap presentation were as for Figure 1. (**A**,**B**) Representative end stage images of vehicle control condition; early Syk inhibition (Syk-IN from start); later Syk inhibition (Syk-IN from 2 min). Images from microspots without (**A**) or with (**B**) TF. Quantitative analysis from ± TF surfaces of parameter P2: thrombus coverage (**Ci**,**Cii**), and P7: fibrin deposition (**Ciii**). Means ± SD, * *p* < 0.05, ** *p* < 0.01, *** *p* < 0.001 vs. indicated group (*t*-test). (**D**) Heatmap of univariate scaled time-dependent values per parameter (P1–7) for indicated surfaces and conditions. (**E**) Subtracted heatmap of scaled parameters versus collagen control runs. Color codes as indicated in color bars. For additional data, see Figure S4.

We also investigated the role of the protein tyrosine kinase Syk as a signaling regulator directly downstream of GPVI. Therefore, we used the inhibitor PRT-060318 (Syk-IN, 20 µM) at a concentration previously shown to completely abrogate the collagen- and GPVI-induced signaling under flow [37]. Examination of end-stage microscopic images showed that the early intervention with Syk-IN caused similar reductions in thrombus size, phosphatidylserine exposure, and fibrin formation (Figure 4A,B), as were observed with anti-GPVI EMF-1 Fab. On either surface (collagen/TF or collagen-only), Syk-IN slightly affected platelet deposition, but it completely abolished the phosphatidylserine exposure and platelet aggregation (Figure S4A,D). Furthermore, in the presence of TF, Syk-IN also annulled fibrin deposition, while all thrombus parameters were strongly downregulated (Figure 4C–E).

Interestingly, the 2 min introduction of Syk-IN did no longer affect the thrombus parameters on collagen/TF microspots. On the other hand, for collagen-only microspots, platelet deposition (P1), the thrombus characteristics (P2–5), and phosphatidylserine exposure (P6) were still reduced at some time points (Figure 4). To sum up, these data confirmed a key role of the GPVI-ITAM signaling pathway involving Syk in mostly the early phase of collagen-induced thrombus formation, whose role extended to later time points only in the absence of TF.

### 2.4. Continued Requirement of Integrin αIIbβ3 Activation in Formation of Platelet-Fibrin Thrombi

**Figure 5:**
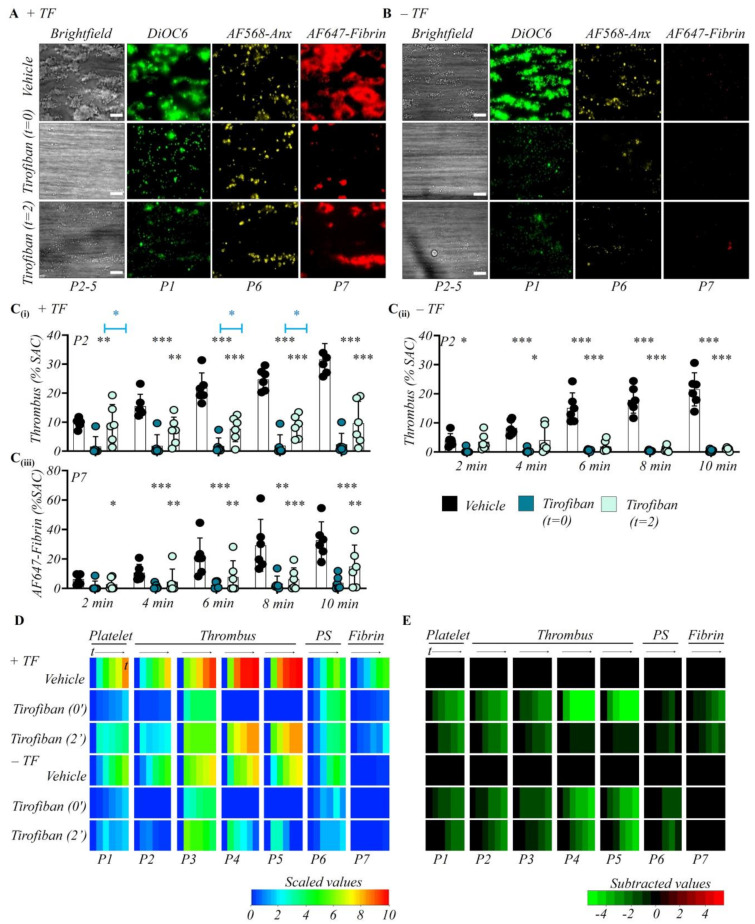
Involvement of integrin αIIbβ3 in thrombus formation independently of tissue factor. Citrated whole blood was labeled and co-perfused with recalcification medium over collagen and collagen/TF microspots (*n* = 6), as for Figure 1. Where indicated, perfusion was switched from control blood to blood preincubated with vehicle or integrin αIIbβ3 inhibitor (tirofiban, 1 μg/mL). Thrombus parameter analysis and heatmap presentation were as for Figure 1. (**A**,**B**) Representative end stage images of (i) vehicle control condition; (ii) early integrin inhibition (tirofiban from start); (iii) later integrin inhibition (tirofiban from 2 min). Images from microspots without (**A**) or with (**B**) TF; bars = 20 μm. Quantitative analysis from ± TF surfaces of parameter P2: thrombus coverage (**Ci**,**Cii**), and P7: fibrin deposition (**Ciii**). Means ± SD, * *p* < 0.05, ** *p* < 0.01, *** *p* < 0.001 vs. indicated groups (*t*-test). (**D**) Heatmap of univariate scaled time-dependent values per parameter (P1–7) for indicated surfaces and conditions. (**E**) Subtracted heatmap of scaled parameters versus collagen control runs. Color codes as indicated in color bars. For additional data, see Figure S5.

Considering that integrin αIIbβ3-mediated platelet aggregation is an essentially reversible event [38], we then monitored the effects of early and late inhibition of αIIbβ3 activation using the integrin antagonist tirofiban (1 μg/mL). As expected, the tirofiban intervention from start completely abrogated the assembly of multi-layered platelet thrombi on collagen microspots, both in the presence and absence of TF (Figure 5A,B). Yet, a monolayer of adhered and spread platelets still formed on either surface type, which illustrated the central role of integrin αIIbβ3 in thrombus build-up but not in flow-dependent platelet adhesion. On collagen/TF surfaces, early tirofiban greatly decreased the thrombus parameters, while phosphatidylserine exposure stayed unaltered and fibrin formation reduced incompletely (Figure 5C–E). On collagen-only surfaces, tirofiban induced similar changes, although it caused a small reduction in phosphatidylserine exposure at 6–8 min, which was related to the lower number of adhered platelets (Figure S5).

Interestingly, the addition of tirofiban after 2 min of blood perfusion led to dismantlement of the thrombi that were formed earlier on the collagen-only surfaces. Multi-layered platelet thrombi were completely gone at end-stage (Figure 5B,C), thus pointing to a retroactive tirofiban effect. On the other hand, on collagen/TF surfaces, the pre-formed thrombi remained intact, but the further thrombus build-up was prevented (Figure 5A,C). In addition, with late tirofiban phosphatidylserine exposure was not affected and fibrin clots were still forming (Figure 5D,E). To sum up, the integrin αIIbβ3 antagonist antagonized the process of thrombus growth from the time point of intervention, and even reversed this process if TF was absent.

## 3. Discussion

**Figure 6:**
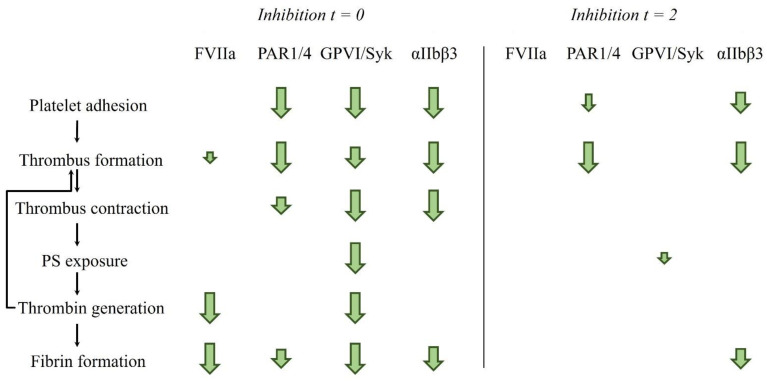
Schematic overview of early and late contribution of platelet and coagulation pathways in thrombus formation on collagen/TF surfaces under flow. Arrow sizes show to which extent inhibition of FVIIa, PAR1/4 receptors, GPVI signaling through Syk kinase, or integrin αIIbβ3 affect the early (0–2 min) and late (2–10 min) stages of the thrombotic process.

In the present study, we used a novel in-house developed intervention method for acutely switching (inhibited) blood samples during perfusion through a microfluidic chamber in order to resolve the time-dependency of platelet activation and coagulation processes via collagen (GPVI-induced ITAM and Syk tyrosine kinase signaling) and TF/thrombin (via factor VIIa and PAR1/4 receptors). As summarized in Figure 6, the results show that all the investigated pathways were crucial for the formation of platelet-fibrin thrombi during the first 2 min of flow. On the other hand, only platelet activation via PAR1/4 and integrin αIIbβ3 activation contributed at later time points. Given that PAR4 [39] and of GPVI [40] have been considered as possible targets for antithrombotic therapy, our results suggest that especially in case of GPVI-dependency an early intervention is important.

Previously, we have demonstrated that the current multiparameter approach of whole-blood thrombus formation under flow over collagen- and TF-coated surfaces provides detailed insights into the importance of specific platelet and coagulation pathways, and that this method can be used as a proxy assessment for hemostasis to explain the bleeding phenotypes of patients with certain platelet or coagulation defects [10,26,41]. In addition, we and others established that collagen-based microfluidic tests can be predictive for the outcome of in vivo models of hemostasis and thrombosis with genetically modified mice [25,42,43]. This background strongly supported us to adapt the microfluidic technology for obtaining information on the time dependency of key pathways of the platelet activation and thrombus-clot formation. For that purpose, we changed the flow perfusion method in a way that pharmacologically inhibited blood samples could enter the microfluidic chamber at a precisely chosen time point. As a suitable interval for separating ‘early’ and ‘late’ events at arterial shear rate of 1000 s^−1^, we choose 2 min, i.e., a time point where the first multi-layered platelet aggregates are formed on collagen-like surfaces [26]. The present results show that separating the process into the first 2 min and later provides good insight into the earlier and later ways of platelet activation.

In the past decades, extensive research has revealed the roles of multiple platelet receptors in the flow-dependent reactions of platelet tethering, adhesion, secretion, aggregation, and coagulant activity (reviewed in [8,9,44,45]). In vivo findings have identified intricate interactions between (TF-induced) thrombin generation and platelet responses to arterial thrombus and clot formation [4,5]. Using the present, modified in vitro flow assay, we can now establish that the roles of GPVI and Syk signaling are most important at earlier stages, such in contrast the roles of platelet thrombin receptors and integrin αIIbβ3, which remained active during a more prolonged time. The present findings are in agreement with studies that in mice the lack of GPVI, obtained via genetic knockout or antibody-mediated depletion, partly protecting from arterial thrombus formation, leaving the generation of unstable, non-contracted thrombi [46,47]. The antithrombotic effects of inhibition of the human platelet PAR1 and PAR4 receptors has also been noticed [48,49]. Already two decades ago, in mice, the deficiency of PAR3 or PAR4 was found to have a thrombo-protective effect [50]. This was also shown for the PAR4 antagonist BMS-986120 when injected into cynomolgus monkeys [39]. These earlier studies hence fit with the current data obtained with pre-incubated blood samples. However, these studies were unsuited to inform on the later temporal roles of these receptors in arterial thrombus formation.

Our data with collagen/TF microspots pointed to an initial role of the extrinsic coagulation pathway via TF and factor VII in thrombin and fibrin formation, seemingly promoted by the procoagulant surface of phosphatidylserine-exposing platelets. This finding also agrees with the literature, underlining the role of platelet phosphatidylserine exposure in thrombus formation [12]. Indeed, the absence of this platelet response in the rare human Scott syndrome leads to a mild bleeding disorder [36]. In atherothrombosis, the initiating role of the TF-factor VIIa pathway has been well studied [51]. In mice, using an in vivo model of atherosclerotic plaque rupture, a study found that inhibition of factor VIIa reduced the arterial thrombus size at early stages [52]. However, whether the exposed TF had a time-restricted role was not investigated.

In recent years, fibrinogen and fibrin are also known as ligands for GPVI during thrombus growth [30,53]. Flow experiments pointed to a sustained but weak GPVI-induced signaling that mostly promoted thrombus stabilization [31]. Our present data suggest that a later, fibrin-dependent role of GPVI is yet smaller than the initial collagen-dependent role. Both GPVI- and PAR-mediated activation can have mutually stimulating effects on platelet activation processes [54]. It appeared that the ‘memory’ of platelets for GPVI-induced activation is longer than for PAR-induced activation. Such a difference in signaling length could also explain the presently identified longer contribution of PARs than of GPVI in the thrombus growth on collagen/TF surfaces. This would imply that the time-confined action of thrombin on platelets necessitates a continued cleavage and activation of these protease-activated receptors.

An interesting observation was the similarly ranged but slightly lower effect of GPVI blockage (with EMF-1 Fab) as compared to Syk inhibition (with Syk-IN). An additional effect of Syk-IN of thrombus formation can have two causes. On the one hand, the EMF-1 Fab cannot block the contribution of other platelet receptors that signal via the Syk kinase, i.e., CLEC-2 and FcγRIIa. A role of CLEC-2 in murine thrombus formation has been postulated [55]. This seemed to be redundant to that of GPVI, as double-deficient mice showed an increased protection from thrombosis and incremented bleeding risk [56]. Since no tool is available to block human CLEC-2, we could not investigate this possibility. On the other hand, we cannot exclude that the EMF-1 Fab still allowed residual GPVI action via a second epitope for collagen binding [57].

A prominent finding was the continued and leading contribution of integrin αIIbβ3 activation (inhibited by tirofiban) to thrombus growth, multilayering, and contraction. This points to a regulatory contribution of αIIbβ3-mediated platelet–platelet contacts during the whole period of thrombus formation and stabilization. Notably, with tirofiban present, the ability of platelets to expose phosphatidylserine was unaltered, albeit this response was reduced in absence of TF due to a lower platelet adhesion. On collagen-only surfaces, we noted that tirofiban but not GPVI blockage had a retroactive effect by inducing destabilization of the formed platelet aggregates. The destabilizing effect was typically lost in the presence of TF, which can be explained by a counter-effect of early formed cross-linked fibrin fibers in the thrombi [20]. Our findings thus mirror the antithrombotic in vivo capacity of αIIbβ3 inhibition by tirofiban and other integrin antagonists [22].

## 4. Materials and Methods

### 4.1. Materials

Recombinant human TF was purchased from Dade-Behring (Breda, The Netherlands), and collagen type I (Horm) was purchased from Takeda (Hoofddorp, The Netherlands). Alexa Fluor (AF)568-labeled annexin A5 was obtained from Life Technology (Carlsbad, CA, USA). The platelet probe 3,3′dihexyloxa carbocyanine iodide (DiOC_6_) came from AnaSpec (Fremont, CA, USA); AF647-labeled human fibrinogen was obtained from Molecular Probes (Eugene, OR, USA). PAR1 inhibitor atopaxar hydrobromide (E5555) was obtained from Axon Medchem (Groningen, The Netherlands). The PAR4 inhibitor BMS-986120 was obtained from Cayman Chemical (Ann Arbor, MO, USA). The anti-human GPVI Fab fragment EMF-1 was generated by Emfret Analytics Würzburg, Germany (unpublished data September 2021). The selective Syk inhibitor PRT-060318 (Syk-IN) came from Bio-Connect (Huissen, The Netherlands). The integrin αIIbβ3 inhibitor tirofiban and bovine serum albumin (BSA) were obtained from Sigma-Aldrich (St. Louis, MI, USA). Pluronic was obtained from Invitrogen (Carlsbad, CA, USA). Other materials came from previously described sources [26,58].

### 4.2. Blood Donors and Blood Collection

Blood was donated by healthy volunteers that were free from anticoagulant or antiplatelet medication for a minimum period of 4 weeks. Studies were approved by the local Medical Ethics Committees (Maastricht University Medical Centre). All donors provided full informed consent in accordance with the Declaration of Helsinki, and procedures were in accordance with the local regulations and guidelines. Blood drawing was by venipuncture using a vacuum container. The blood was collected into a 9 mL tubes with 3.2% trisodium citrate (Greiner, Alphen a/d Rijn, The Netherlands). For all the microfluidics studies, collected blood was stored at room temperature and used within 4 h.

### 4.3. Preparation of Microspot Coatings

Glass coverslips (24 × 60 mm, Thermo-Fisher, Breda, the Netherlands) were cleaned and degreased and subsequently freshly coated with the help of a precision mall with collagen as two adjacent microspots (≈1 mm of diameter and 5 mm center-to-center distance), of which the downstream spot contained TF, as detailed elsewhere [26]. To prevent a cross-over effects, we placed the most thrombogenic microspot (collagen/TF) as second in the direction of blood flow. Coating was performed with 1 μL of collagen type I (50 µg/mL), and, after 1 h with extra 1 μL TF (500 pM), including a washing step in between. After another incubation with HEPES buffer (pH 7.45; 2 mM MgCl_2_, 0.1% glucose, 10 mM Hepes, 136 mM NaCl, and 2.7 mM KCl), the coverslips were blocked using HEPES buffer containing 1% BSA (w/v). Subsequently, these were mounted on a transparent flow chamber (50 μm height, 3.0 mm width and 30 mm length) and pre-rinsed with HEPES buffer at a pH of 7.45 with 0.1% BSA added [20].

### 4.4. Recalcification and Mixing of Blood Samples under Flow in Microfluidic Chambers

Citrate-anticoagulated blood samples were in situ recalcified while perfusing through the flow chamber under conditions of full blood-buffer mixing, essentially as described before [26], but using a three-way mixing tube system (Figure S1). Two citrated blood samples in 1.0 mL plastic syringes were connected to two of the tube inlets and sequentially perfused through the flow chamber using pulse-free micro-pumps (Model 11Plus, 70–2212, Harvard Apparatus, Holliston, MA, USA). The third tube inlet was connected to a 1 mL syringe containing recalcification medium with 63 mM CaCl_2_ and 32 mM MgCl_2_ in HEPES buffer with a pH of 7.45 (see Figure S6). Mixing of either blood sample with recalcification medium was at a volume ratio of 10:1. Flow rates were set to provide a final calculated final wall-shear rate of 1000 s^−1^ [59]. Blood samples for the secondary perfusion were pre-incubated with indicated inhibitor for 10 min at room temperature. As a standard procedure, both the primary and secondary blood samples were pre-labeled with final concentrations of 0.5 μg/mL DiOC_6_ (staining platelets), 8.5 μg/mL AF647-fibrinogen (staining of fibrin accumulation), and 4.0 μg/mL AF568-annexin A5 (staining phosphatidylserine-exposing platelets). Note that discriminative staining for detection strongly labeled fibrin vs. low-labeled fibrinogen was by threshold settings, as detailed elsewhere [20]. To facilitate the access of Syk-IN into the platelets, we pre-mixed the compound PRT-060318 in DMSO with pluronic (40 µg/mL), and then it was added to the blood [37]. The concentration of DMSO was kept at <0.5%. Per blood sample and condition, duplicate or triplicate (in case of variation) flow runs were performed.

Complete mixing of the blood samples with the medium for re-calcification was achieved by a two-phased mix procedure [26]. First, a three-way-shaped Versitec silicone tubing (1.0 mm ID, 3.0 mm OD, Saint-Gobain Plastics, France), fabricated in house, was built by a dual cross-wise needle insertion into the middle of a long portion of tube (at opposite sides). Two other tubes were then glued onto the central tube while removing the needles. This created face-to-face openings in the middle tube, which allowed a free passage of fluids. Liquid polymerizing silicone was used to provide leakage-free sealing. The second phase of mixing occurred when the central tube outlet entered the flow chamber inlet, which resulted in the rapid transition from a tubular (1 mm Ø) to a flat (width 3 mm and height 50 μm) cross-section, and consequently in an acute redistribution of the flow velocity profile [26]. This set up ensured the continuous and consistent mixing with a low shear rate at the inlet, while keeping a laminar shear flow inside of the parallel-plate chamber. Full mixing was visually inspected by microscopy (absence of separate streams of blood and recalcification medium).

By usage of two connected syringes with blood, it was possible to initially perfuse an untreated blood sample (2 min) and then to switch to a treated blood sample, with the third syringe pump with constant running of recalcification buffer. This operation procedure allowed for an immediate switch from untreated to treated blood without stasis, and preventing mixing of the blood samples. All flow runs were performed with the three-tube system.

### 4.5. Microscopic Real-Time Detection of Multicolor Thrombus Formation on Microspots

Microscopic brightfield and three-color overlay imaging of thrombus formation on two microspots was performed, essentially as described [26]. In brief, fluorescence and brightfield microscopic images were acquired by rapid switching of dichroic cubes (brightfield, or filter sets with excitation wavelengths 626 nm (Cy5), 531 nm (RFP), and 470 nm (GFP)). An inverted EVOS fluorescence microscope was used to record images (Life Technology, Ledeberg, Belgium). The microscope was equipped with an Olympus 60 × oil-immersion objective with high *z*-axis resolution (UPLSAPO60, numerical aperture 1.35). The images were collected as 8-bit monochromes by a sensitive camera, providing 1360 × 1024 pixels and a resolution of 0.108 μm per pixel. Per flow run, image sets were collected at t = 2, 4, 6, 8, and 10 min.

### 4.6. Standardized Microscopic Image Analysis and Assessment of Thrombus Parameters

Brightfield and fluorescence images were analyzed with the help of semi-automated scripts, designed in the open-access program Fiji [60]. Specific scripts were designed for brightfield images and for each fluorescent label, essentially as described [61,62]. In these scripts, optimized fast Fourier transformation was applied to filter down the image-wide structures and reduce background noise. Afterwards, morphological horizontal and vertical erode and dilate steps were applied to remove the noise and to enhance relevant structures. In order to verify the binary masked images, overlay images were generated. In case of incorrect overlays, scripts allowed to loop back for the resetting of thresholds. Per microspot and time point, the analyzed fluorescence and brightfield images resulted in seven parameters (P1–7) (Table 1). Fluorescence DiOC_6_ images reported on platelet deposition (P1), and enhanced brightfield images showed thrombus surface area coverage (P2). Furthermore, brightfield images were scored for thrombus morphology (P3): 0, no or little attached platelets; 1, multiple single attached platelets; 2, widespread coverage of single attached platelets; 3, small platelet aggregates; 4, medium-sized aggregates or thrombi; 5, big aggregates or thrombi [61]. In addition, scoring (scale 0–3) for thrombus contraction (P4) and thrombus multilayering (P5). Other fluorescence images provided quantitative information on phosphatidylserine-exposing platelets (P6, AF568-annexin A5); furthermore, on fibrin formation (P7, thresholded AF647-fibrinogen).

### 4.7. Data Handling and Statistics

Heatmaps were generated using the program R. For heatmap representation, all parameter values were univariate-normalized to a range of 0–10 [61]. To obtain one parameter set per microspot and donor, we averaged thrombus parameters values of duplicate or triplicate flow runs from the same blood donor. In order to visualize treatment effect, we linearly subtracted scaled parameters in order to obtain subtraction heatmaps. For each inhibition time-point (t = 0 and t = 2 min), differences between mean values of control and inhibitor runs were determined between treated and untreated (vehicle control) blood samples per donor using a paired Student’s *t*-test. *p*-values below 0.05 were considered to be significant. In addition, for the subtraction heatmaps, a filter of *p* < 0.05 was added to visualize relevant effects where indicated. Numerical data of interventions are presented as means with SD.

## 5. Conclusions

In conclusion, this study presents a new efficient adaptation of the Maastricht microfluidic system, allowing disclosure of the time-dependent roles of key pathways of platelet activation and coagulation in the thrombus development. Our first-time study on the interference in thrombus formation at different time points has revealed typical differences between early and late inhibition (Figure 6). Our data indicate that, in the microfluidic setting, GPVI-induced platelet signaling is pivotal only in the early phases of thrombus growth, although this signaling can continue to induce phosphatidylserine exposure in later phases. Furthermore, it appears that thrombin induction via PAR1/4 continues to be functionally active in thrombus growth for a longer time-period as compared to GPVI-induced signaling. Finally, integrin αIIbβ3 activity is operative during all phases of thrombus formation. The present results thereby provide a significant addition to the current understanding of the sequence of molecular processes in arterial thrombus formation. 

## Figures and Tables

**Table 1:** Parameters and microscopic image sources of thrombus formation. Seven parameters were obtained per whole-blood flow run at time points 2, 4, 6, 8, and 10 min. These were categorized into platelet (P1), thrombus (P2–5), and coagulation-related parameters (P6–7). Abbreviations: PS, phosphatidylserine; SAC, surface area coverage.

Parameter	Time (min)	Image Type	Description	Unit
Platelet parameter				
P1	2–10	DiOC_6_	platelet adhesion	% SAC
Thrombus parameters				
P2	2–10	brightfield	thrombus coverage	% SAC
P3	2–10	brightfield	thrombus morphology	score 0–5
P4	2–10	brightfield	thrombus contraction	score 0–3
P5	2–10	brightfield	thrombus multilayering	score 0–3
Coagulation parameters				
P6	2–10	AF568-annexin A5	platelet PS exposure	% SAC
P7	2–10	AF647-fibrin(ogen)	fibrin deposition	% SAC

## Data Availability

All data are included in the manuscript as figures, tables, or supplementary figures.

## Supplementary Materials

The following supporting information can be downloaded at: https://www.mdpi.com/article/10.3390/ijms23010358/s1.
