# Supplementary material for: Temporal Roles of Platelet and Coagulation Pathways in Collagen- and Tissue Factor-Induced Thrombus Formation
Source: Int J Mol Sci. 2021 Dec 29;23(1):358. doi: 10.3390/ijms23010358 (PMC8745329; doi:10.3390/ijms23010358)
Supplement: Supplementary file 1 [file ijms-23-00358-s001.zip › ijms-1505203-supplementary.pdf]

Online supplement

Temporal roles of platelet and coagulation pathways in collagen and tissue factor induced thrombus formation

Stefano Navarro [1,2], David Stegner [1], Bernhard Nieswandt [1], Johan W.M. Heemskerk [2,3] and Marijke J. E. Kuijpers [2,4,*]

[1] Department of Vascular Biology, Rudolf Virchow Center for Experimental Biomedicine, Würzburg Josef-Schneider-Straße 2, 97080 Würzburg, Germany.

[2] Department of Biochemistry, Cardiovascular Research Institute Maastricht (CARIM), Maastricht University, 6229 ER Maastricht, The Netherlands

[3] Synapse Research Institute, Kon. Emmaplein 7, 6214 AC Maastricht, The Netherlands. E-mail: jwmheem722@outlook.com

[4] Thrombosis Expertise Center, Heart and Vascular Center, Maastricht University Medical Center+, Maastricht, Professor Debyelaan 25, 6229HX Maastricht, The Netherlands. Tel. +31-43-3881674. E-mail: marijke.kuijpers@maastrichtuniversity.nl

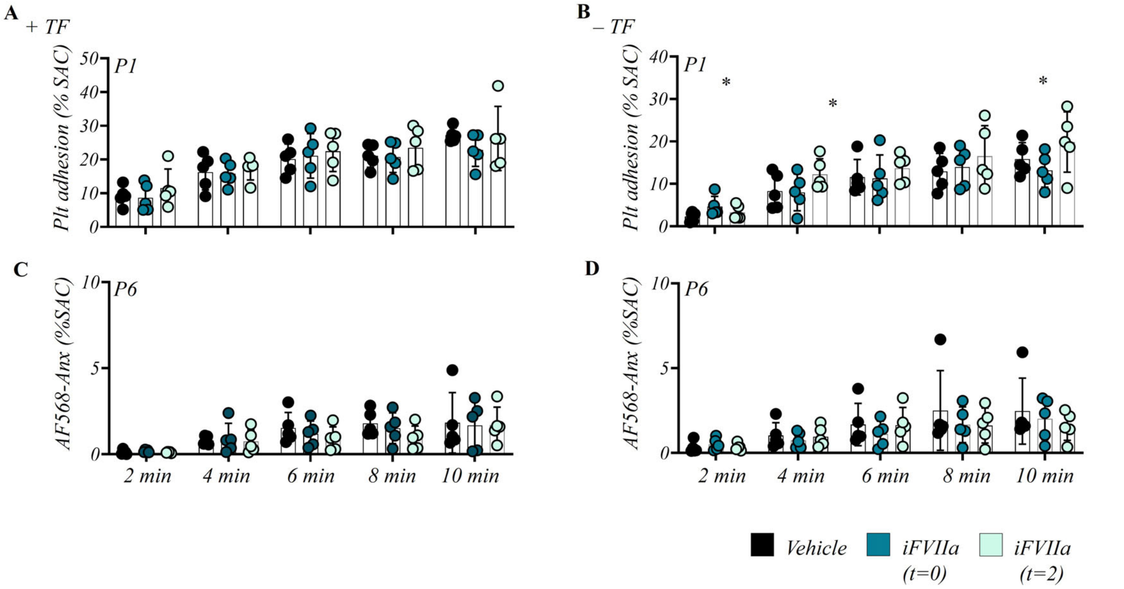

Figure S1. Priming role of TF in progression of platelet-fibrin thrombus formation on collagen under high shear. Citrated whole blood from healthy subjects (*n* = 5) was supplemented with fluorescent labels to simultaneously detect platelet adhesion (P1, $DiOC_6$), thrombus and platelet multilayer characteristics (P2-5, brightfield), phosphatidylserine exposure (P6, AF568-annexin A5) and fibrin deposition (P7, AF647-fibrin). Where indicated, perfusion was from start (t=0) with iFVIIa-treated blood (1 µM, f.c.), or the control blood was switched after 2 min with iFVIIa-treated blood (t=2). Control blood runs were carried out with vehicle solution. During blood flow, monochromatic images in 4 colors were captured from collagen/TF and collagen microspots by microscopy at 2, 4, 6, 8 and 10 min. Shown are quantitative effects of iFVIIa on thrombus parameters: (**A**, **B**) platelet adhesion (P1); and (**C**, **D**) AF568-annexin A5 staining for phosphatidylserine exposure (P6). Means ± SD, *p <0.05, **p <0.01, ***p<0.001 *vs.* indicated group (t-test). See further Figure 1.

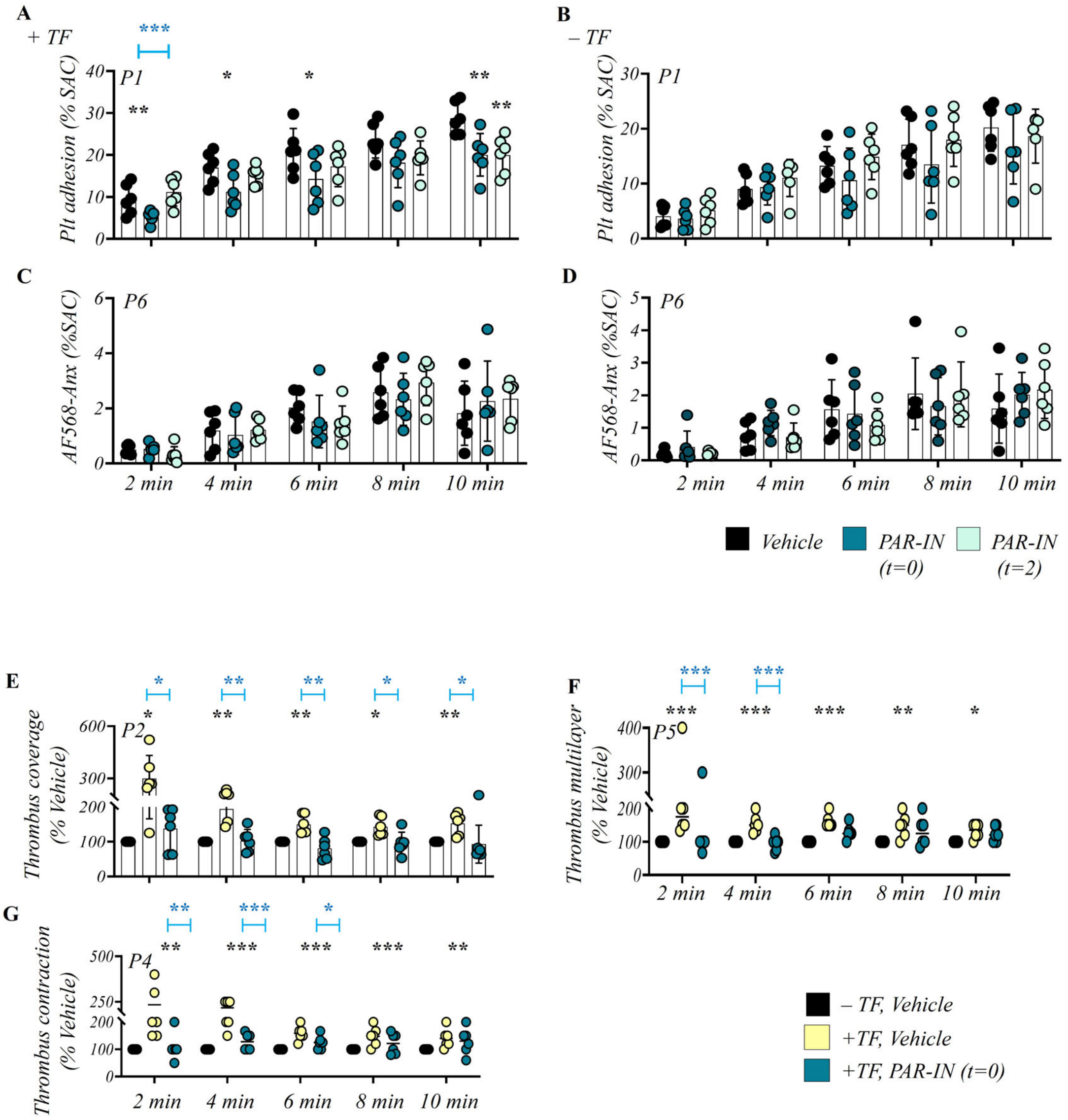

Figure S2. Temporal roles of platelet thrombin receptors PAR1 and PAR4 in platelet-fibrin thrombus formation on collagen with TF. Citrated whole blood samples from healthy subjects (n = 6) were supplemented with fluorescent labels, and perfused over microspots of collagen and collagen/TF, as described for Figure 1. Where indicated (PAR-IN), perfusion was switched from control blood to blood preincubated with vehicle or a mix of atopaxar (PAR1 inhibitor, 2 μM, f.c.) and BMS-986120 (PAR4 inhibitor, 1 μM, f.c.). Shown are quantitative effects of PAR-IN on thrombus parameters: (**A**, **B**) platelet adhesion (P1); and (**C**, **D**) AF568-annexin A5 staining for phosphatidylserine exposure (P6). Furthermore, normalized (*vs.* -TF vehicle) quantification per donor of effects of +TF vehicle and PAR-IN on: (**E**) thrombus coverage (P2); (**F**) thrombus multilayering (P5); and (**G**) thrombus contraction (P4). Means ± SD, *p <0.05, **p <0.01, ***p <0.001. *vs.* indicated group (t-test). See further Figure 2.

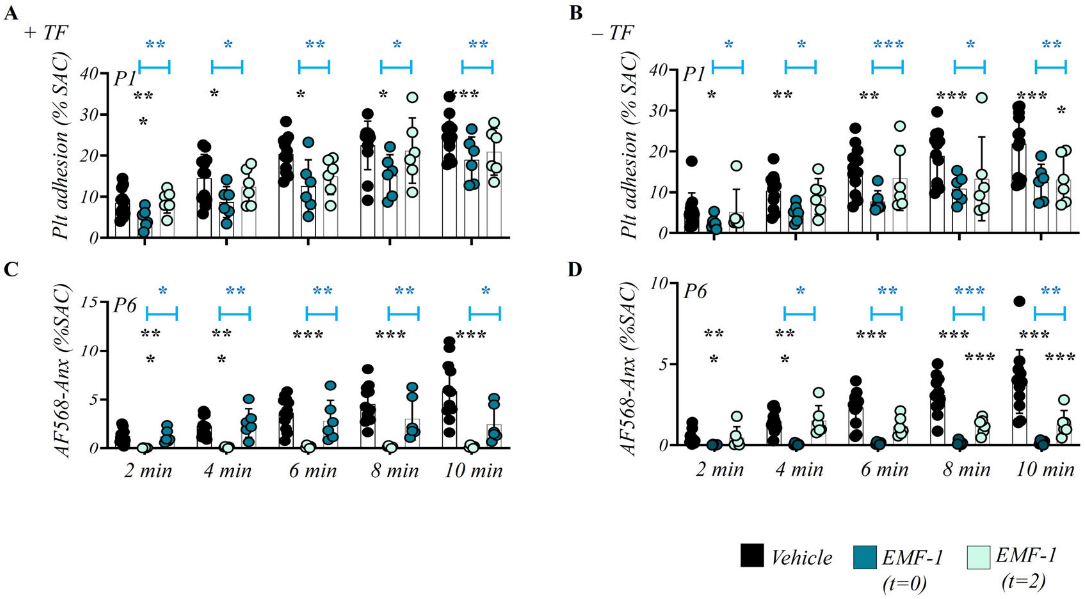

Figure S3. Time-dependent role of GPVI receptor in platelet-fibrin thrombus formation on collagen. Citrated whole blood was labeled and co-perfused with recalcification medium over collagen and collagen/TF microspots (*n* = 6), as for Figure 1. Where indicated, perfusion was switched at 2 min from control blood to blood preincubated with vehicle or anti-GPVI Fab EMF-1 (10 μg/mL, f.c.). Shown are quantitative effects of EMF-1 Fab on thrombus parameters: (**A**, **B**) platelet adhesion (P1); and (**C**, **D**) AF568-annexin A5 staining for phosphatidylserine exposure (P6). Means ± SD, *p <0.05, **p <0.01, ***p <0.001 *vs*. indicated group (t-test). See further Figure 3.

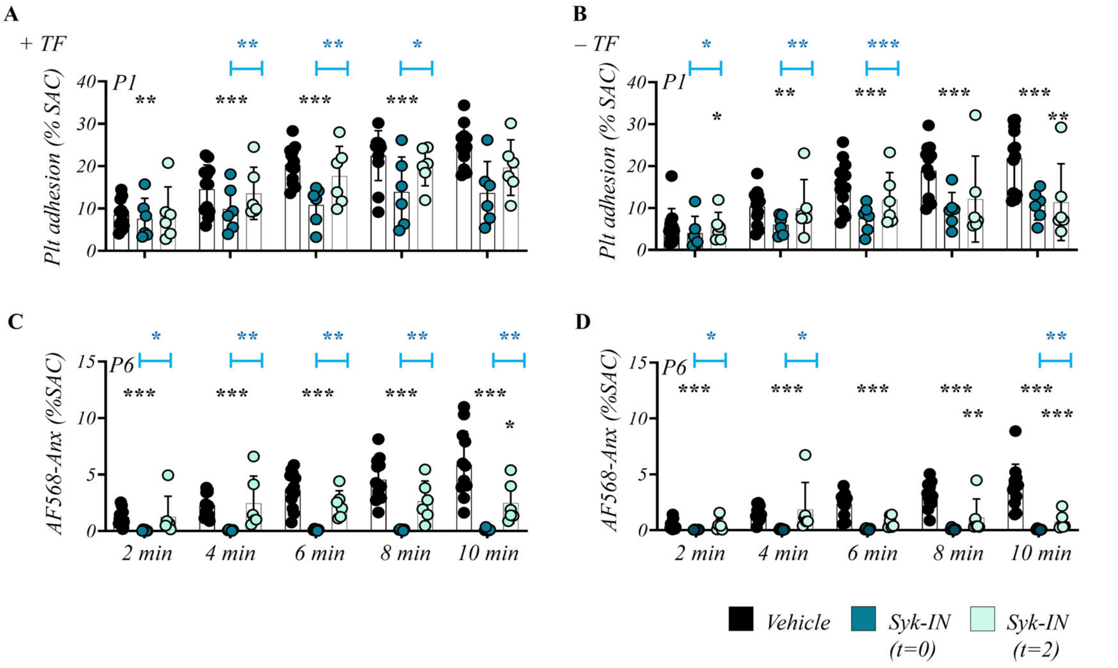

Figure S4. Temporal role of platelet Syk activation in platelet-fibrin thrombus formation independently of TF. Citrated whole blood was labeled and co-perfused with recalcification medium over collagen and collagen/TF microspots(n = 6), as for Figure 1. Where indicated, perfusion was switched at 2 min from control blood to blood preincubated with vehicle or Syk-IN (PRT-060318, 20 μM). Shown are quantitative effects of Syk-IN on thrombus parameters: (**A**, **B**) platelet adhesion (P1); and (**C**, **D**) AF568-annexin A5 staining for phosphatidylserine exposure (P6). Means ± SD, *p <0.05, **p <0.01, ***p <0.001 *vs*. indicated group (t-test). See further Figure 4.

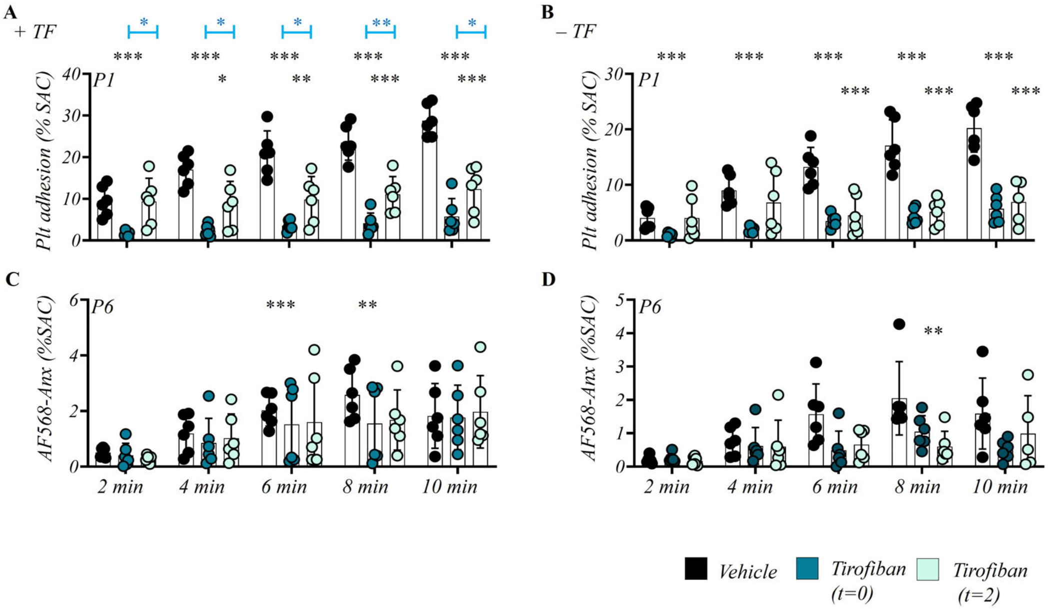

Figure S5. Persistent role of integrin αIIbβ3 in platelet-fibrin thrombus formation independently of tissue factor. Citrated whole blood was labeled and co-perfused with recalcification medium over collagen and collagen/TF microspots (*n* = 6), as for Figure 1. Where indicated, perfusion was switched from control blood to blood preincubated with vehicle or integrin αIIbβ3 inhibitor (tirofiban, 1 µg/mL). Shown are quantitative effects of tirofiban on thrombus parameters: (**A**, **B**) platelet adhesion (P1); and (**C**, **D**) AF568-annexin A5 staining for phosphatidylserine exposure (P6). Means ± SD, **p* <0.05, ***p* <0.01, ****p* <0.001 *vs*. indicated group (t-test). See further Figure 5.

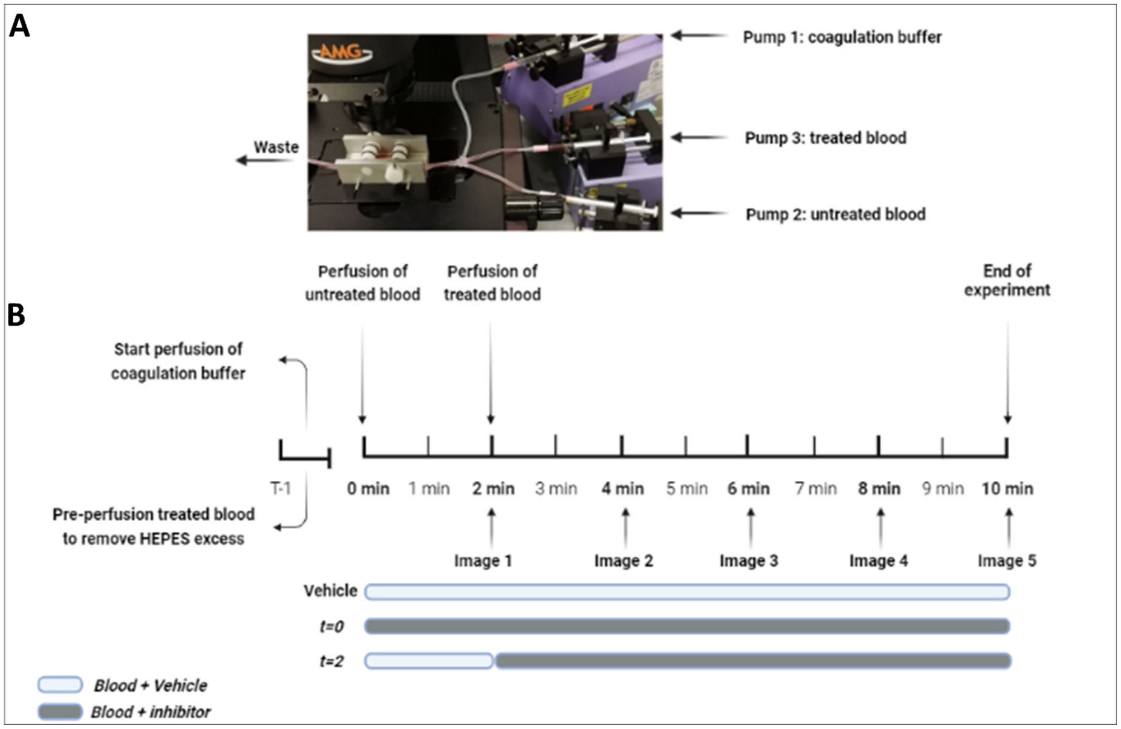

Figure S6. Setup and flowchart of three-way tubing inlet system to for immediate switching from vehicle control blood to inhibited blood. (**A**) Picture of three connected tubes, allowing pressure infusion of recalcification medium into respective blood samples. The syringe attached to pump 1 contains recalcification medium with 32 mM $MgCl_2$ and 63 mM $CaCl_2$ in Hepes buffer pH 7.45, the syringe attached to pump 2 contains blood labeled with $DiOC_6$, AF568-annexin A5 and AF647-fibrinogen and vehicle, and the syringe attached to pump 3 contains blood with the same labels plus required inhibitor. (**B**) Flowchart showing the sequence of actions during an experiment. Pump 1 operated constantly at 1x flow rate. Before start of the experiment, pump 3 was run at 9x flow rate to allow removal of Hepes buffer pH 7.45 from the connection tube. The pump 3 was stopped once the blood reached the connection point. Pump 2 was run at 9x flow rate from start during the first 75 s, after which pump 3 took over. Of note, the time required for blood from the connection point to the inlet of the flow chamber was timed at 45 s (final calculated wall-shear rate of 1000 s^{-1}). This timing thus allowed perfusion for the first 2 min of untreated blood and consecutively of treated blood for the next 8 min.
